# Supplementary material for: What does the mean mean? A simple test for neuroscience
Source: PLoS Comput Biol. 2024 Apr 19;20(4):e1012000. doi: 10.1371/journal.pcbi.1012000 (PMC11062559; doi:10.1371/journal.pcbi.1012000)
Supplement: S1 Supplementary Methods — We detail all the relevant parameters and tools we made use of. (PDF) [file pcbi.1012000.s001.pdf]

S1 Supplementary Methods

The Supplementary Methods are divided into three sections:

1. **Decoders**
2. **PCA analysis**
3. **Data Clustering**

Decoders

In order to select the most informative brain areas for Data set 2 in a data-driven way, we made use of different decoders. For each experimental session, there are several recorded regions. Thus, we trained independent decoders using the single-trial population vector for each region. The labels to be predicted would be either choice (left wheel turn, right wheel turn or no movement) or stimulus (right-higher contrast, left-higher contrast, both equal).

We split the data following a $80-20$ ratio (train-test). Given the imbalanced nature of the dataset, we used a stratified (i.e., ensuring that all classes are fairly represented across each fold) $10-$repeated $5-$fold Cross-Validation to fine-tune each model's hyperparameters using a Bayesian approach and checked that the model performance was above majority class (i.e., always predicting the most abundant label) and random models.

In the following, let assume we want to classify N input variables (x_i, with $i = 1, ..., N$) into target variables that can belong to K classes (i.e., $y_i \in \{1, ..., K\}$).

Bayesian optimization

Hyperparameter optimization is arguably one of the bottlenecks when deploying Machine Learning techniques. Fortunately, in the last years, the field is rapidly evolving and we now have access to quick and intuitive libraries that alleviate greatly these tasks. One of these libraries is *Optuna* [1]. We relied on Tree-structured Parzen Estimators (TPEs) [2] to speed up hyperparameter search.

For each model, we chose different hyperparameters to be optimized, which we will detail in the following descriptions. We relied on the SciKit-Learn package in Python [3] to implement all of the classifiers.

Gaussian Naive Bayes

Naive Bayes assumes independent features [4]. In terms of the covariance matrices, this model assumes they are diagonal. Particularly, the Gaussian Naive Bayes (GNB) model assumes that the class-conditional densities are normally distributed as:

$$P(\mathbf{x}|y = c, \mu_c, \Sigma_c) = \mathcal{N}(\mathbf{x}|\mu_c, \Sigma_c) \tag{1}$$

where μ and Σ are the class-specific mean vector and class-specific covariance matrix, respectively. In order to compute the class posterior, we can simply make use of Bayes' theorem

$$P(y = c|\mathbf{x}, \mu_c, \Sigma_c) = \frac{P(\mathbf{x}|y = c, \mu_c, \Sigma_c)P(y = c)}{\sum_{k=1}^{K} P(\mathbf{x}|y = k, \mu_k, \Sigma_c)P(y = k)}. \tag{2}$$

So, in order to classify $\mathbf{x}$ into a class c:

$$\hat{h}(\mathbf{x}) = \underset{c}{\text{argmax}}\ P(y = c|\mathbf{x}, \mu_c, \Sigma_c) \tag{3}$$

For this classifier, we did not perform any hyperparameter tuning.

k-Nearest Neighbors

This model relies on the assumption that the closer two points are, the more similar they are and the more likely it is that they belong to the same class. It is implemented as follows: for any given point x_i, compute its distance to the rest of the points; then, select the k points that are closest; out of those, apply a majority vote rule. As a summary: the most prevalent class of the k closest points to x_i will determine the class that we predict for it.

For this classifier, we optimized the number of nearest neighbors and the leaf size of the k-d Tree algorithm.

Random Forest

This model makes use of several Decision Trees (DTs) to solve supervised learning problems. DTs are non-parametric models that split the data using a given number (depth) of conditional steps - populated with at least with some number of data points (minimum sample split). When we aggregate several DTs (using a technique known as bootstrap aggregating or *bagging*), we end up with a *Random Forest* (RF). Specifically, we sample with replacement from the data and feed these subsamples to different trees. Finally, we use a majority vote for each tree's output as the RF prediction. This procedure reduces the variance in the model (i.e., mitigates overfitting) but has a minimal effect on its bias (i.e., underfitting is still a risk). Therefore, ideally we would like to use DTs that are unstable (high variance) but very little bias. In order to check the model performance, we compute its out-of-bag (oob) error - cases in the training data that are not in a significant part of the bootstrapped samples.

For this model, we optimized the number of DTs, their maximum depth and the minimum sample split.

Generalized Linear Model

We trained a multinomial Generalized Linear Model (GLM) with a L2-regularization. This model states that the probability of a particular data point y_i belonging to class c is given by:

$$p\left(y_i = c \mid x_i\right) = \frac{e^{w_c \cdot x_i + b_c}}{\sum_{j=1}^{K} e^{w_j \cdot x_i + b_j}} \tag{4}$$

After having the probabilities of y_i belonging to each class c, the highest one will be taken to be 1 and the rest will be set to 0. Therefore, the objective is to find the weight vector w_c that minimizes the distance between the predicted $(\hat{y}_i)$ and the actual (y_i) class labels by optimizing (in this case, minimizing) the following loss function:

$$L\left(\hat{y}_i, y_i\right) = -\log\left(\frac{e^{w_c \cdot x_i + b_c}}{\sum_{j=1}^{K} e^{w_j \cdot x_i + D_i}}\right) + \lambda \left\|w_c\right\|_2^2 \tag{5}$$

where $\|w_c\|_2^2$ is the L2-norm of the weight vector for class c, accounting for the L2-regularization term, with λ modulating its strength. The inverse of λ $(C = 1/\lambda)$ is the hyperparameter we optimized for this model.

Support Vector Machines

This class of models rely on finding the hypersurface that maximizes the separation between classes in the data. To this end, it is important to first find the *support vectors* (SVs), which are the closest ones to the surface. There are two main types of Support Vector Machines (SVMs): linear SVMs and kernel-based SVMs. The difference is that, in the former, the separating hypersurface is a hyperplane (i.e., a non-curved hypersurface), while, in the latter, it is is allowed to be curved. Mathematically, the *linear* version of this model is the solution to the following optimization problem:

$$\min_{\omega_i \in \mathbb{R}^N} \quad \mathcal{C}(\omega_i) \tag{6}$$

where

$$\mathcal{C}(\omega_i) = C \sum_{i=1}^{N} \overbrace{\max\big(0, 1 - y_i x_i\big)}^{\text{Hinge loss}} + \overbrace{||\omega||^2}^{\text{L2-reg.}} \tag{7}$$

In order to arrive to the equivalent one for the non-linear SVM, we can take advantage of the fact that this problem satisfies some conditions [5] that allow us to construct its dual form. It takes the form of:

$$\mathcal{C}_D(\alpha_i) = \sum_{i=1}^{N} \alpha_i - \frac{1}{2} \sum_{i=1}^{N} \alpha_i \alpha_j y_i y_j K(\mathbf{x_i}, \mathbf{x_j}), \quad \text{with} \quad 0 \leq \alpha_i \leq C, \sum_{i=1}^{N} \alpha_i y_i = 0 \tag{8}$$

where $K(\mathbf{x_i}, \mathbf{x_j})$ is the kernel (it can be linear or not) and C is the regularization strength. In this work, we chose Radial Basis Functions (RBF) as our kernel. These are given by:

$$K(\mathbf{x_i}, \mathbf{x_j}) = \exp\big(-\gamma ||\mathbf{x}_i - \mathbf{x}_j||^2\big) \tag{9}$$

where γ is the reach parameter (i.e., how far we want two separate points influence each other).

For these decoders, we optimized the regularization strength (C) and, only for the *RBF*, the reach coefficient (γ).

Mutual Information

After having trained each decoder, we separately computed the Mutual Information between the predicted and the test class labels, as a proxy of the amount of stimulus – or choice – information there was in the population vector.

This quantity is defined in the context of classical Information Theory [6, 7] and we can compute it for two discrete stochastic variables X and Y. Assuming these have a joint probability mass function given by $p_{X,Y}(x, y) = P(Y = y \mid X = x) \cdot P(X = x)$ and that each of them follows a marginal probability distribution given by $p_X = \sum_{y \in Y} p_{X,Y}(x, y)$, one can mathematically define the Mutual Information between X and Y as:

$$I(X;Y) = \sum_{x \in X} \sum_{y \in Y} p(X,Y) \log \left(\frac{p_{X,Y}(x,y)}{p_X p_Y} \right) \tag{10}$$

Intuitively, one can understand $I(X;Y)$ as the uncertainty reduction in X that follows if Y is measured (or vice versa, as $I(X;Y)$ is invariant when swapping X and Y). If (and only if) they are independent of each other, then $I(X;Y) = 0$. Therefore,

this is a strictly non-negative quantity. It is noteworthy that $I(X;Y)$ captures all linear and nonlinear dependencies between X and Y, thus generalizing the notion of correlation measures. For further discussion of this measure, see [8, 9].

Hierarchical clustering

After having computed the resulting Mutual Information for all areas, sessions and decoders, we aimed to check the stability of the selection of the most informative brain areas, so that results were not highly dependent on which model we used to choose them. To do that, we used an unsupervised method, known as hierarchical clustering. Particularly, our vectors were the aggregated information (stimulus + choice) that each decoder extracted, over all brain areas. Once we have the pairwise distance between points (proximity matrix), this method can be understood in an iterative manner: merge the closest points in a cluster, then merge the closest clusters and repeat until only a single cluster (encompassing all points) remains. We used the Euclidean metric to compute the proximity matrix.

To implement this algorithm, we relied on the Seaborn [10] (Python package) implementation of *clustermap*.

Elbow method

In order to select a threshold when selecting the task-related areas based on their stimulus and choice information, we used the data to compute the Kernel Density Estimate, via Gaussian kernels [11]. After having extracted these, we used the method discussed in [12] to find the point of maximum curvature. We made use of the kneed Python package, implemented by the same authors [12].

PCA Analysis

Templates and distances in PCA space

As an alternative to Pearson's correlation, we applied Principal Component Analysis (PCA) [13]. We chose PCA over non-Negative Matrix Factorization [14] or other more advanced dimensionality reduction techniques such as LFADS [15] or PSID [16] because we wanted to keep all analyses as general as we possibly could. Thus, we computed the truncated Singular Value Decomposition (tSVD) [17] for the matrix consisting of Z-scored single-trial population vectors, for a given area and session. Then, we extracted the knee (elbow) using the aforementioned method, to select the number of components based on the variance explained. After the number of components has been selected, we projected each single-trial into this (dimensionally-reduced) space and computed the Euclidean distance between this new vector and the template (also projected into this space). We normalized by the distance between the projection of the two templates in this new space.

Specificity Index for PCA distances

Since in PCA analyses we dealt with distances rather than correlations (i.e., differences rather than similarities), we inverted the computation of the Specificity Index in this context so that positive values continued to signify a stronger relation between single trial response and correct template than incorrect template. The corresponding formula is:

$$\rho_i^{PCA} = \frac{d(\lambda_{wrong}^{PCA}, r_i^{PCA}) - d(\lambda_{correct}^{PCA}, r_i^{PCA})}{d_{ref}} \tag{11}$$

where d stands for Euclidean distance and r_i^{PCA} is the PCA-projected version of the population vector measured in the ith trial; λ_{wrong}^{PCA} and $\lambda_{correct}^{PCA}$ are the PCA-projected version of the trial-average templates (wrong and correct, respectively). Finally, $d_{ref} = d\big(\lambda_{wrong}^{PCA} - \lambda_{correct}^{PCA}\big)$, which we took to be the reference distance.

Data Clustering

Clustering by Pupil size

As a way to account for the animal's behavioral state, we grouped together trials that had a similar pupil size. Firstly, we low-pass filtered the recorded pupil size (that came as an output of DeepLabCut [18]) using a Butterworth filter [19] of order 4. We chose to filter out any frequency above $1Hz$, as we were interested in slow variations in pupil size, which have been linked to attentional state [20, 21]. We then computed the mean pupil size in the same time window we used for the neural analyses (200 ms after stimulus presentation). Finally, we ranked all sizes over trials and grouped them in brackets of 10 percentiles (e.g., trials with a pupil size between the 32th percentile and the 42th one would be grouped). Within the selected group, we repeated the template-matching algorithm that we used in the main text, tailoring the average to those trials that shared a similar pupil size.

k-Means clustering

In order to group trials according to similarity in the recorded neural response, we used k-Means clustering. This algorithm, after randomly initializing k centroids (one per cluster), is as follows: (1) Compute the distance between each data point and these k centroids. (2) Each point will belong to the cluster with the closest centroid. (3) New centroids will be given by the actual points belonging to a cluster.(3) Repeat until convergence (when centroids move no more). As this is an unsupervised method, we used the elbow method to select the number of k centroids in which to cluster the data. Finally, as we did for the pupil size grouping, we repeated the template-matching algorithm that we used in the main text, tailoring the average to those trials that belonged to the same cluster.

References

1. Akiba T, Sano S, Yanase T, Ohta T, Koyama M. Optuna: A next-generation hyperparameter optimization framework. In: Proceedings of the 25th ACM SIGKDD international conference on knowledge discovery & data mining; 2019. p. 2623–2631.

2. Bergstra J, Bardenet R, Bengio Y, Kégl B. Algorithms for hyper-parameter optimization. Advances in neural information processing systems. 2011;24.

3. Pedregosa F, Varoquaux G, Gramfort A, Michel V, Thirion B, Grisel O, et al. Scikit-learn: Machine learning in Python. the Journal of machine Learning research. 2011;12:2825–2830.

4. Domingos P, Pazzani M. On the optimality of the simple Bayesian classifier under zero-one loss. Machine learning. 1997;29(2):103–130.

5. Rockafellar RT. Convex analysis princeton university press. Princeton, NJ. 1970;.

6. Quiroga RQ, Panzeri S. Extracting information from neuronal populations: information theory and decoding approaches. Nature Reviews Neuroscience. 2009;10(3):173–185.

7. Shannon CE. A mathematical theory of communication. The Bell system technical journal. 1948;27(3):379–423.

8. Timme NM, Lapish C. A tutorial for information theory in neuroscience. eneuro. 2018;5(3).

9. Cover TM, Thomas JA. Information theory and statistics. Elements of Information Theory. 1991;1(1):279–335.

10. Waskom ML. seaborn: statistical data visualization. Journal of Open Source Software. 2021;6(60):3021. doi:10.21105/joss.03021.

11. Silverman BW. Density Estimation for Statistics and Data Analysis. Monographs on Statistics and Applied Probability. 1986;26.

12. Satopaa V, Albrecht J, Irwin D, Raghavan B. Finding a" kneedle" in a haystack: Detecting knee points in system behavior. In: 2011 31st international conference on distributed computing systems workshops. IEEE; 2011. p. 166–171.

13. Pearson K. LIII. On lines and planes of closest fit to systems of points in space. The London, Edinburgh, and Dublin philosophical magazine and journal of science. 1901;2(11):559–572.

14. Lee DD, Seung HS. Learning the parts of objects by non-negative matrix factorization. Nature. 1999;401(6755):788–791.

15. Pandarinath C, O'Shea DJ, Collins J, Jozefowicz R, Stavisky SD, Kao JC, et al. Inferring single-trial neural population dynamics using sequential auto-encoders. Nature methods. 2018;15(10):805–815.

16. Sani OG, Abbaspourazad H, Wong YT, Pesaran B, Shanechi MM. Modeling behaviorally relevant neural dynamics enabled by preferential subspace identification. Nature Neuroscience. 2021;24(1):140–149.

17. Hansen PC. The truncatedSVD as a method for regularization. BIT Numerical Mathematics. 1987;27(4):534–553.

18. Mathis A, Mamidanna P, Cury KM, Abe T, Murthy VN, Mathis MW, et al. DeepLabCut: markerless pose estimation of user-defined body parts with deep learning. Nature neuroscience. 2018;21(9):1281–1289.

19. Butterworth S, et al. On the theory of filter amplifiers. Wireless Engineer. 1930;7(6):536–541.

20. Hoeks B, Levelt WJ. Pupillary dilation as a measure of attention: A quantitative system analysis. Behavior Research methods, instruments, & computers. 1993;25(1):16–26.

21. Kang OE, Huffer KE, Wheatley TP. Pupil dilation dynamics track attention to high-level information. PloS one. 2014;9(8):e102463.
