# Supplementary material for: What does the mean mean? A simple test for neuroscience
Source: PLoS Comput Biol. 2024 Apr 19;20(4):e1012000. doi: 10.1371/journal.pcbi.1012000 (PMC11062559; doi:10.1371/journal.pcbi.1012000)
Supplement: Attachment — Submitted filename: 2024-01 Response to reviewers PLOS CompBio.pdf [file pcbi.1012000.s016.pdf]

Response to Reviews:

What does the mean mean? A simple test for neuroscience

Alejandro Tlaie, Katharine Shapcott, Thijs van der Plas, James Rowland, Robert Lees, Joshua Keeling, Adam Packer, Paul Tiesinga, Martha Havenith, Marieke Schölvinck

General Remarks

Both reviewers found this work interesting and relevant for the readership of *PLoS Computational Biology*. The main issues they identified were in the argumentation and general readability of the paper. We thank the reviewers for their valuable comments, which have substantially improved the manuscript, and we hope that our response addresses the issues raised by the reviewers in a satisfactory manner. All changes in the manuscript are marked in bold.

Response to Reviewers

Reviewer 1

Tlaie and colleagues investigate novel criteria for defining brain areas as relevant for a task: reliability of the responses and a relationship between single-trial consistency and behavioral responses. The authors use two datasets for their case study, and analyze these datasets rigorously and in depth. Dataset 1 passes the criteria but dataset 2 does not pass these criteria because, the authors claim, the information is dynamically encoded. However, I think this is an example of the short-comings of the criteria, particularly criterion 1. I discuss the caveats of the approach below, and note some potential discussion points.

We thank the reviewer for their thoughtful comments and suggestions, which have been very helpful in clarifying the arguments made in the paper.

The single-trial responses are in most cases positively correlated to the trial-averaged responses to some extent. The close-to-zero correlations observed on many trials are consistent with low firing rates with Poisson variability.

We agree with the reviewer that low firing rates are one prominent source of low trial-to-trial reliability. Our approach accounts for this factor by incorporating the following two steps: 1) Instead of absolute single-trial correlations, we examine the difference between single-trial correlations to the correct and to the incorrect averaged response. If an individual trial features low firing rates, this would presumably affect the correlation to all cross-trial averages equally, and thus shouldn't impact the difference

between them (i.e. the specificity index). We have **clarified the description of this point in the main text (Lines 128-131)**. 2) We compare all single-trial correlations and specificity indices to surrogate data (see e.g Fig. 2C and Fig. 4D), which have been bootstrapped by generating the same number of spikes as featured in the actual trial. Specifically, as detailed in Fig. S3, for each recorded trial our bootstrapping procedure assumes a Poisson process generating the same number of spikes, and allocates each spike to a neuron with a probability given by the trial-averaged template. In this way, our bootstrapping procedure accounts for differences in correlation that would arise solely from low spike numbers if neuronal activity adhered to a Poisson process. We realize that this was not stated sufficiently clearly in the manuscript, and **have amended text and figure legends to reflect the Poisson assumptions of our surrogate data (see Lines 199-207, 431, as well as 109-111, the legends of Figs. 2C and 4D, and the Surrogate Models subsection in Methods).**

Alternatively, one extreme example I can think of where the correlation would be low is if there are two populations of neurons that are driven by a given stimulus on average, but on any given stimulus one set of these stimulus-selective neurons is on and the other is suppressed somewhat. Both of these cases do not reduce the ability of a downstream area to decode information: a downstream brain area can decode the stimulus by pooling the stimulus-driven neurons' inputs.

We thank the reviewer for bringing up this example, which is a great illustration of how our test should be operating. We agree that it is entirely possible for the same stimulus to be encoded by different but internally reliable neuronal populations across different trials. We would also argue that this is a prime example of a situation where simply averaging neuronal responses across all trials would be a distinctly suboptimal representation of the true neuronal dynamics, since it would obscure the alternating neuronal response patterns. Our criteria are designed to reflect that: A cross-trial average taken across all trials would receive a low Specificity Index, which in this case would point at the fact that it does not accurately reflect either of the distinct neuronal response patterns. If, on the other hand, one were to first cluster trials according to similarity, and then compute cross-trial averages only within those trial clusters, they would receive a much higher Specificity Index, indicating that these within-cluster averages are a better representation of the underlying neuronal activity.

In fact, this is exactly what our analysis of agnostically clustering trials into groups based on similarity showed for Data Set 2 (Fig. 6B): The Specificity Index increased markedly when computing averages within trial clusters, suggesting a similar coding scenario to the one the reviewer mentions. In contrast, trial clustering did not improve specificity in Data Set 1. In this case, our criteria served to highlight that in Data set 1, responses are more uniform across all trials, while in Data set 2, there seem to indeed exist two different 'response modes' that should be treated individually when computing averages. Our criteria can be used to reveal such unique trial structures, and that would very much be one of the purposes we hope they will be applied for in future. Based on the reviewer's comments, we feel that this is a crucial point to clarify in order to help readers apply our test in the most useful ways. We have therefore **added an extended explanation to the Discussion of how our criteria might operate in different neuronal coding scenarios (see Lines 309-318 and 485-492)**.

This for example is the function of the “super-neurons” in (Stringer et al 2021), to pool over many neurons, not to choose the “super-coder” neurons. Indeed, the authors found that the behavioral relevance criterion increases as a function of the number of neurons considered.

Also, in (Perez-Ortega et al 2021) neuron groups are found using unsupervised clustering (not directly using stimulus-driven properties), and there is no mention of finding “super-coder” neurons; instead they discuss the stability of these groups of neurons across days.

We thank the reviewer for bringing up this fascinating point - we have also wondered if we could find any such ‘super-coder neurons' based on averaged firing rate responses. Specifically, we hypothesized that even if for instance visual brain areas did not show particularly stereotyped single-trial responses, or if information was represented in a highly distributed way (see below), downstream ‘super-neurons’ directly involved in behavioural decision-making should extract information in the way the reviewer describes, and thereby arrive at more stable responses, reflecting either stimulus identity or behavioural choice robustly. Data set 2 is particularly well suited to test this notion, given the large number of neurons recorded across a wide range of brain regions. If there are down-stream ‘super-coder neurons’ that are reading out information e.g. from more distributed representations in upstream areas, then they can likely be captured in such a data set.

The supplementary analysis shown in Fig. S9 was our first approach towards exploring this possibility explicitly. In this analysis, we implemented a jackknifing approach, where we removed neurons individually from the population and quantified the resulting drop in the Specificity Index of the population responses. We reasoned that, if a brain region contained particularly robust readout neurons (i.e. ‘super-neurons’), then the drop in response specificity after jackknifing those neurons should be particularly large. As shown in this figure, distributions of ΔSpecificity (before-after jackknifing), were close to symmetric in most brain areas. As a result, the skewness parameter γ of the ΔSpecificity distribution approaches 0. This indicates that there was no distinct population of particularly powerful readout neurons.

However, a few brain regions featured a somewhat right-skewed distribution ($\gamma > 0$), meaning that for these areas, the Specificity Index decreased more sharply when certain neurons were removed, suggesting that there were indeed some detectable ‘super-neurons’. Even more intriguingly, the contribution of such super-neurons to the overall response specificity was stronger in hit trials than in miss trials. This suggests that in trials when the responses of these ‘super-neurons’ were more specific, animals tended to act more successfully, which in turn hints that ‘super-neuron’ responses did indeed contribute to successful task performance. The areas highlighted in this analysis are also consistent with the reviewer’s arguments, given that they mainly cover hubs of visual, motor and reward processing. Specifically, our ‘super-neuron’ analysis highlighted six brain areas: The red nucleus (RN, a subcortical hub for motor coordination), the subparafascicular thalamic nucleus (SPF, auditory processing), the primary visual cortex (VISp, visual processing), reticular substantia nigra (SNr, reward and motor planning) and midbrain reticular nucleus (MRN, arousal and conscious state). Interestingly, these areas are overwhelmingly subcortical. This might either point to the fact that task performance in this paradigm was driven mainly by subcortical computations, or that subcortical areas are more likely to develop ‘stereotyped’ neuronal responses that are represented more accurately by cross-trial averages.

In Data Set 1, we found no evidence of super-neurons altogether ($\gamma \approx 0$). This may reflect the fact that task information is distributed more evenly throughout the neuronal population, or that this data set did not contain recordings from brain areas that might contain 'super-neurons'. We had omitted these more in-depth arguments regarding Fig. S9 in the previous manuscript for the sake of brevity, but based on the reviewer's feedback, we believe they add to the understanding of our test in important ways, so we have **added them to the main text of the paper (Lines 242-265 and 457-464).**

Beyond the question of super-neurons, we also agree with the reviewer that in general, simply taking more neurons into account is likely to lead to a much better approximation of the computations carried out in any given neuronal population. As the reviewer points out, this was already somewhat evident from the extrapolation analysis in Fig. 6, where at least the behavioural relevance of cross-trial averages does increase with higher neuron numbers. **We have added several statements in the Results and Discussion to point out this effect (Lines 236-238, 271-277 and 464-471).**

To explore this point further, we have also added a new analysis, in which we select either the top or the bottom 10% of most/least informative neurons (as given by their jackknifed Specificity Index described in the previous paragraphs). We then plot how Specificity Index and Behavioral Relevance change when only including these most/least informative neurons, compared to the metrics achieved when taking into account the entire neuronal population.

As shown in the **new Fig. S10** (see below), the Specificity Index unsurprisingly increases when selecting the most informative neurons only, rather than the whole population. Interestingly, it only decreases slightly for the least informative neurons compared to the overall population. However, there is no consistent improvement in Behavioural Relevance when we select either the most or least informative neurons. If anything, the strongest decreases in Behavioural Relevance occurred when focusing only on the most informative neurons. In other words, behaviour is better predicted by the responses of a large, mixed population of neurons, than by the responses of a smaller group of particularly selective neurons. This finding speaks in favour of the reviewer's notion that to understand neuronal computations we must consider the population at large, not each individual neuron. We have **added this analysis as the new Fig. S10, and also summarized these findings and their interpretation in the Results and Discussion sections (Lines 271-277 and 464-471).**

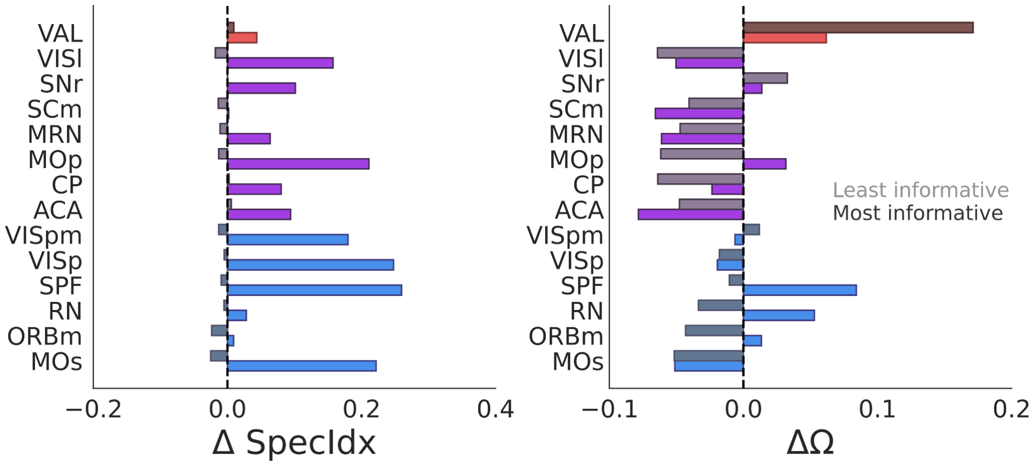

A case where the decoder *would* fail is if the trial-to-trial variability was information-limiting (Moreno-Bote et al 2014). For example, if in response to the left grating, all the neurons responded on some trials like the grating was on the right, then the stimulus could not be decoded from the population on those trials. A neural population can be unreliable but not have these detrimental correlations.

We couldn't agree more with the reviewer that the brain features many different forms of 'variability' or rather dynamic change, many of which do *not* impair information read-out and may in fact support it. One of the reasons why we devised the test presented here is that standard metrics like cross-trial averages by their very nature pool all these different types of variability into one category: unexplained variance or 'noise'. Especially in contexts where more than one source of variance over time is present (which likely applies to many cases of neuronal information processing), we would argue that it would therefore be better to use metrics that are able to differentiate different types of variability rather than ignoring them. To give a simple example, our Figure 7 shows that performing a Principal Component Analysis before cross-trial averaging increases both Specificity and Behavioural Relevance in Data set 2, but not in Data set 1. This would suggest that specifically in Data set 2, such an approach helps to eliminate variance that is obscuring information available at the single-trial level. As such, our test can help to clarify under which circumstances a standard approach is largely warranted, and where it fails to capture the underlying structure of neuronal activity, including its different forms of dynamic change.

To elaborate on these points, we have **added an extended description comparing the outcomes of different analyses approaches shown in Figure 7** to the Results section, together with interpretations why the same analysis might have different effects in Data set 1 and 2 (Lines 329-344). In the Discussion, **we have also added a section on how the utility of cross-trial averages is affected by different types of variance**, both in terms of their covariance with stimulus-related dynamics, and in terms of their time signatures (e.g. state-dependent switches between distinct response modes versus slow response shifts due to e.g. learning or representational drift) (see Lines 507-515 and 485-492).

Indeed using dataset 2 the authors claim that motor and visual areas are "barely reliable and behaviorally relevant", but inactivation of these areas leads to the mouse being unable to perform the task (Zatka-Haas et al 2021). Thus, the claim that these measures, particularly reliability, relate to task performance is unproven.

We completely agree with the reviewer that e.g. visual and motor areas are absolutely needed to perform the task used in Data set 2. These areas are clearly involved in processing crucial task-related information - and they do so very successfully, given that the resulting behavioural responses are overwhelmingly correct! This is why it is particularly interesting that the cross-trial averages of neuronal firing rates in these areas appear to be so ill-suited to represent neuronal activity in individual trials.

In our view, there are two explanations for this striking disconnect: 1) Average population firing rates might be the 'wrong' neuronal metric to average. To give a completely hypothetical example, if instead of firing rates, one instead averaged the phase-locking of individual neurons to theta oscillations across trials, those cross-trial averages might be perfectly representative of stimulus identity and/or behavioural choice at the single-trial level. 2) The idea of treating responses as fundamentally stationary (which is inherent to cross-trial averaging) does not apply correctly to this data set.

This does not mean that the relevant information cannot be decoded in other ways - in fact we would argue that it definitely can. After all, our goal with this paper is not to decode task-related information in the best possible way, but rather to test how optimal or suboptimal the field's current default analysis approaches are. Because of this, we have focused on the most popular and simple approaches (Firing Rates, their Z-scored versions, PCA, etc.) that are commonly applied across different subfields of neuroscience. Based on these extremely common approaches, our paper demonstrates that in Data set 2, cross-trial averages of firing rates are a suboptimal way to represent the neuronal information that is clearly being conveyed by the brain areas in question. As such, the low performance of Data Set 2 on both our criteria would suggest that, at least in this data set, other analysis approaches should be sought out, that do the conveyed neuronal information better justice. **Some of the options for such more promising approaches are highlighted in the Discussion (Lines 537-577).**

I still think these criteria are very useful, just with some of these caveats mentioned. For example, it might be useful to discuss how increased numbers of neurons improve the behavioral relevance, suggesting that sampling more neurons per brain area can influence criterion 2. Also, it might be useful to discuss that certain types of unreliability are more detrimental than others to decoding analyses.

We agree that discussing these points in greater depth is crucial so that potential applications of our tests in different data sets become clearer. We have therefore extended the Results and Discussion to elaborate on these points, including an **extended interpretation of our extrapolation to higher neuron numbers (Fig. 6; Lines 235-238 and 465-467), of our simulation results (Lines 369-370, 384 and 516-531), as well as a longer analysis of how different neuronal coding schemes - and sources of cross-trial variability - would affect our test metrics (Lines 486-492, 457-471 and 507-515)**.

Reviewer 2

In their manuscript, Tlaie and co-authors embark on an exciting crusade to uncover the mean and meaninglessness of the trial-averaged view of population activity. They propose two central measures to capture how informative the averages are to characterize population activity and to predict behavioral outcomes. They start from the first principles:

1. If means are informative, they shall be well correlated with the population activity at each trial, and more so, they should be more correlated with the average activity of the same trial condition than with the different one.

2. If the upstream population (or researcher) is using means as a perfect template for the behavioral choice, closeness to the mean should be predictive of the quality of the behavioral response.

The authors derived the measures to capture how well these two principles are realized in the recorded data and evaluated them on two datasets. Interestingly, they found that in one dataset (with more constrained experimental conditions), the means were a rather good description of the population activity. In contrast, in the less constrained conditions, they were uninformative.

I generally liked the paper: the question is relevant and timely, the methods are solid and imaginative, the images are great, and the writing is quite clear.

We thank the reviewer for their encouraging and very helpful feedback on our paper.

I have some moderately minor and some really minor comments.

1. To me, it feels that results are sometimes stated stronger than what is visible from the images/ analysis. Specifically, in the discussion of 5B and C, the authors state in 208-209, “in Data set 2, the relation between single-trial responses and trial-averaged 209 templates is neither reliable nor specific.” However, in the figure and discussion, they need to use arguments that the indices/differences are significant but not large enough. While I support the usage of effect sizes, I feel they do not warrant, in this case, such a strong statement.

We thank the reviewer for pointing this out, and **have amended both the specific phrasing and similar phrases throughout the text (see for example Line 236-237).**

2. Further on, figure 7A: Here, I am not sure if the expected specificities of different data pre-processing are of the same ranges. In any case, it would be interesting to read why the reliability of the z-scored firing rate is larger than for Firing rates in Data2 and smaller in Data 1, and if the reliability of some data pre-processing is so large, is it still reasonable to say that the means are not informative for data2?

The reviewer is raising an important point here. To our understanding, Z-scoring improves Specificity in Data Set 2 but not Data Set 1 for the following reason: In Data Set 1, the experimenter is directly stimulating some of the neurons and spontaneous firing rates are rather low; in the other case, the stimulus is more subtle, inter-trial variability is larger, and baseline firing rates vary more strongly. Z-scoring neural responses over trials seems to increase Specificity in Data Set 2 by readjusting neural variability to the same baseline, thereby preventing the response of some neurons with higher baseline firing from obscuring the response of others. We have **added explanations of why the same preprocessing steps might lead to different outcomes in the two data sets to the Results and Discussion (Lines 329-344).**

3. A similar problem seems to appear in the discussion of 6C: 280: “… grouping trials by average pupil size did not improve specificity”. However, panel 6C on the Specificity index clearly shows an increase, even if possibly not sufficient. Even more so for 6B and specificity (we expect it to grow because of picking the more correlated neurons, but the statement seems to contradict the picture).

We thank the reviewer for highlighting this discrepancy, and **have amended our descriptions of these effects to be more representative of the data (see Lines 309-318 and 322-323).**

4. The part on the surrogate data model is hard to read, the main text didn’t call the distribution (Beta distribution) and didn’t reference the methods. For Fig 8 it is unclear where the squares indicating data comes from.

We thank the reviewer for pointing these issues out; **the manuscript has been updated to incorporate their solutions and generally clarify the structure of the surrogate data model (see additions in Lines 355-372 and the caption of Fig. 8B).**

It refers to the function as Gaussian (299), but it just looks bell-shaped, it has a constrained domain so cannot be Gaussian.

We appreciate the reviewer's attention to detail. Indeed, the Beta distribution does not exactly equate the Gaussian distribution for any set of parameters. However, as can be shown [see https://www.math.wm.edu/~leemis/chart/UDR/PDFs/BetaNormal.pdf], in the case of $\lim_{\alpha \to \infty} \beta(\alpha, \alpha)$, the Beta function converges to the standard normal distribution.

In the original text we stated that diverging values of this parameter "indicate an increasingly tight unimodal Gaussian function" in order to simplify the language somewhat. We have now **rephrased this description to be more mathematically accurate and have included the appropriate reference (see Lines 356-358).**

In the suppl Fig. it does not look like the experimental data is symmetric, so would not be a uni-parametric Beta distribution. Did you fit Beta to responses? How was SNR evaluated for the data?

In both cases, what we did was to compare the experimental values (Specificity Index and Behavioral Relevance) with the simulation ones. For those parameter combinations where these values were compatible, we added the squares. We did this because the model we used is a simplified one that allows us to get an intuition of the problem we are tackling. Thus, it does not have all the parameters and complexity that the real neuronal activity has; so we are basically 'backwards-inducing' from the type of Indices we got to the types of variability/response structure that might produce such Indices. **We have added a brief explanation of this strategy for clarity (Lines 520-531).**

Some writing comments: The paper is overall very well written. My suggestions are mainly to improve the "reader experience".

6. I was missing the references to the particular parts of the vast method section. I feel they are also necessary because, at least for me, it was challenging to match the section titles with the information I sought. I would suggest adding numbering/lettering to the subsections in methods and referring to them directly.

We thank the reviewer for this suggestion and now **refer to six subsections of the Methods section throughout the main text:** 'Specificity and Behavioural Relevance Index', 'Decoders', 'Surrogate Models', 'PCA analysis', 'Data Clustering' and 'Simulation'.

7. For me, some parts feel a bit bloated, making it harder to get to the (simple and straightforward)

point. Notably, the discussion repeats the results in a rather long form. I would suggest shortening it, especially the parts about checking alternative hypotheses.

We thank the reviewer for pointing this out - we **have shortened the initial description of the results in the Discussion section (e.g. Lines 409-413 and 431-435)**, but added some potential interpretations of our findings based on the feedback from Reviewer 1.

8. Also, in the discussion (366-372), you mention “benchmarking.” This term has a strong meaning in some communities, overlapping with PLOS CB readership (checking the performance on a standardized test and comparing it to the previously presented solutions). I think this meaning does not fit your case. You illustrate the usage of the metrics, but you do not have ground truth or competitor-metrics.

We thank the reviewer for drawing our attention to this. We have **replaced all instances of the term ‘benchmarking’** by terms like ‘example data’.

Minor:

We thank the reviewer for taking the time to provide this very helpful in-depth check of the manuscript. We have **amended all minor issues pointed out here, and updated the manuscript accordingly.**

9. In Fig 2C middle: it seems like the bootstrap is still from the correct trial group. If not then I missed how it is done and why you still have the same value as on the left.

This was indeed a mistake, which we have now corrected in Fig. 2C. We have also **added a clearer explanation of the bootstrapping process in the main text (see Lines 109-11 and 199-207, and captions of Fig 2C and 4D).**

10. For Fig 4B-C, it feels like the thresholds for B were taken such that the areas would end up being exactly the same as in C (at least for the Stimulus Information even the smallest threshold change will add or remove some areas). I think the match is remarkably independent of exact thresholding but maybe the total coincidence invites over-confidence in the results. For better visibility, I would have colored the names of the most informative areas in C in the colors corresponding to their counterparts in B.

We thank the reviewer for pointing this out. The complete match of identified areas across Figs. 4B-C is indeed a coincidence – we used a standard elbow criterion to partition the distributions of the stimulus/choice information per brain area, which is of course fixed, and was decided on before we conducted the hierarchical clustering across decoders to validate the results. To give readers a better chance to evaluate the results for themselves, we **colour-coded the area labels in Fig. 4C as the reviewer suggested, added**

labels also for the areas that did not meet the criteria, and added lines in Fig. 4B to better visualize the elbow criterion.

We also want to point out that the areas highlighted by our decoders were highly representative of all other recorded areas in terms of the relevant indices , i.e. Specificity and Behavioral Relevance (see Fig. S7, reproduced here for convenience). As such, a somewhat different selection of brain areas would be highly unlikely to change the overall conclusions of our analyses.

We have **added these arguments in brief form to the description of the decoder analysis (Lines 172, 181-188)** to make it easier to follow for readers.

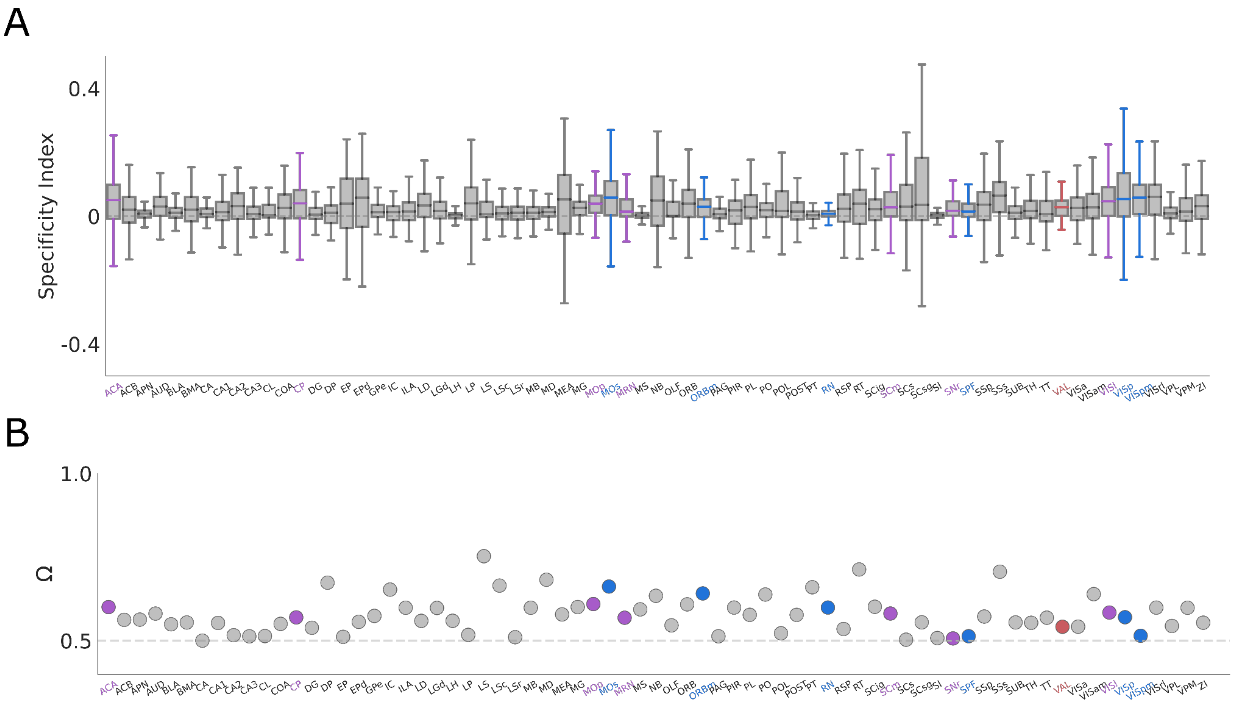

11. Line 187: I think you might have meant S3A

12. 298 and later in the text something happened with “<” and “>”

13. 314 “Data Set 1 should” be distributed

14. In methods: 617 you mean the “Pearson correlation coefficient”.

15. In Behavioral relevance part would be good to tell what are n_X, n_Y

16. Eq 17: k in {0,1} (you need brackets for the correct notation)

17. Fig S12. plotting (as, e.g., non-filled circles) the Omega for raw firing rates would make your statement visible

18. Fig S13 lacks the caption.
